# Mesh-associated complications in minimally invasive ventral mesh rectopexy: a systematic review

**DOI:** 10.1007/s00464-024-11369-7

**Published:** 2024-11-08

**Authors:** Gabriel Fridolin Hess, Fabio Nocera, Stephanie Taha-Mehlitz, Sebastian Christen, Marco von Strauss Und Torney, Daniel C. Steinemann

**Affiliations:** 1 Clarunis, University Digestive Health Care Center, St. Clara Hospital and University Hospital Basel Postfach, 4002 Basel, Switzerland; 2 University of Basel, Medical Faculty, Basel, Switzerland

**Keywords:** Rectopexy, Rectal prolapse, Mesh, Obstructive defecation

## Abstract

**Background:**

Ventral mesh rectopexy (laparoscopic and robotic) is a common and well established treatment of rectal prolapse. Although described as safe and effective, complications, especially mesh-associated ones are often mentioned. Additionally, there is no consensus regarding the mesh type and fixation method as well as the materials used for this purpose. The aim of this systematic review was to identify the total amount of complications and of those the mesh-associated ones.

**Methods:**

Pubmed, Web of Science and Cochrane Central Register were screened for complications in general and in detail regarding the mesh(es) and a systematic review was performed.

**Results:**

Following qualitative evaluation, 40 studies were identified for further investigation. Across 6269 patients, complications were found in 9.2% (622 patients). Mesh-related complications were described in 1.4% (88 patients) of which 64.8% were erosions, 11.4% fistulas and 13.6% mesh releases. The complication rate according to the different materials were low with 1% in biological and synthetic meshes and 1.8% in not further described or mixed mesh type. Non-absorbable material to fixate the mesh was most frequently used to fixate the mesh.

**Conclusion:**

Laparoscopic ventral mesh rectopexy is a safe operation with a low-complication rate, regardless of mesh type.

Rectal prolapse (RP) due to posterior pelvic floor weakness, is a life-impairing problem. Up to 90% of the patients are women [1]. Aetiology is multifactorial, including chronic constipation, vaginal delivery, previous pelvic surgery, heavy lifting, obesity and age, the aetiology is multifactorial [2]. Various problems such as bleeding, soiling, incontinence, constipation, formation of rectal ulcers and metaplasia can occur. RP can be categorised by the Oxford classification (I-V) in which distinction is made between internal and external, as well as different expressions of the intussusception [3]. When conservative therapy using laxatives, nutritional therapy and physiotherapy fail, surgical treatment is indicated in internal rectal prolapse. External rectal prolapse is considered a relative indication for surgery. The current most popular approach, described by D’Hoore, is laparoscopic or robotic anterior or ventral mesh rectopexy (LVMR/RVMR) [4]. The literature shows tendencies that the minimally invasive approach is preferable to the open technique especially with regard to the short-time outcomes [5]. However, a clear consensus regarding the best technique is not yet described in the literature [6–9]. In some health systems like the NHS in United Kingdom, ventral mesh rectopexy (VMR) is a highly debated topic and under *“high vigilance restriction”* [10]. Furthermore, the United States Food and Drug Association (FDA) warns of mesh erosions up to 4% in the first 23 months after surgery [11]. Given these public concerns and reports, pelvic floor surgeons have reported on mesh-related complications in a number of prospective and retrospective studies in the past. A total of three systematic reviews of the available literature have been published since 2013 addressing the mesh-related complications with limited patient numbers and conflicting or unclear results concerning the role of mesh type and other contributing factors such as the type of mesh fixation [12–14].

The present systematic review includes all available publications and a larger number of patients with further analysis of contributing factors for mesh complications such as the type of fixation.

## Methods

The present systematic review was prepared in accordance to the Preferred Reporting Items for Systematic Reviews and Meta-Analyses (PRISMA) guidelines [15]. Inclusion criteria, search strategies and endpoints were defined in advance. The study was registered on PROSPERO (CRD42023423962).

### Literature search strategy, study selection and data collection

A representative literature search was conducted using the electronic databases of Pubmed, Web of Science and Cochrane Central Register of Controlled Trials, and studies published up to the 31th of March 2024 were considered. Search parameters were: *(mesh rectopexy) AND (ventral rectopexy)*. The common literature understands a minimally invasive, especially laparoscopic (LVMR) and nowadays more often robotic (RVMR) procedure under VMR mesh rectopexy. Only minimally invasive operations were considered. The reference lists of previous systematic reviews and meta-analyses also were reviewed to identify potentially relevant articles. Two investigators (GFH and FN) performed data extraction, quality assessment and critical appraisal. Disagreements were resolved through a third reviewer (DCS).

Data from the included studies was entered in an Excel™ (Microsoft Corporation, Redmond, Washington, USA) database. A double data-entry method was used to avoid errors in data extraction. Data was compared and discussed by the two investigators regarding discrepancies until a consensus was achieved.

### Eligibility criteria

Data extraction was performed by the two authors independently considering titles and abstracts of the articles for eligibility. The inclusion criteria were as follows: (1) a prospective or retrospective study design; (2) surgical treatment of rectopexy with a mesh; (3) patient age > / = 18 years. Accordingly, the following exclusion criteria were also used: (1) no reports relating to mesh complications; (2) were not written in English; (3) reported as part of the same study population; (4) the article was published as a case report, review article, letter to the editor, editorial, or conference abstract; (5) cohort under 10 participants.

### Outcome measures

The main outcomes analysed were postoperative complications, separated into overall and mesh-specific complications. Mesh complications include erosions, fistulas, mesh release (detachment/separation from the underlying fascia), discitis and complications not described in detail. Furthermore, a differentiation was made between minor and major complications. A major complication was defined as Clavien Dindo IIIb or higher [16]. In studies where no differentiation between minor and major was made, the complications were reviewed by the two investigators (GFH and FN) and allocated to minor and major where applicable.Any discrepancies were discussed with a third reviewer (DCS). In 10 studies, a distinction between minor and major complications was not possible, i.e. some studies subdivided the patients between surgical and medical complications.

Secondary outcome measures were type of suture material utilised and mesh types in relation to complications. Additionally, papers were screened for operation details such as redo-surgeries after a prior rectopexy repair, laparoscopic or robotic approach and combination surgery.

### Methodological quality

To evaluate the methodological quality of the randomised controlled trials (RCTs), the Cochrane Collaboration tool was used to assess the risk of bias. Random sequence generation, allocation concealment, blinding of participants, personnel and outcome assessment, incomplete outcome data, selective reporting and other sources of bias were considered. For each domain, the risk of bias was classified as low, unclear or high according to the Cochrane Handbook for Systematic Reviews of Interventions [17]. Non-randomised trials were rated according to the Newcastle–Ottawa quality assessment scale for case–control studies [18]. Up to nine stars could be awarded: selection of study groups (maximum 4 stars); comparability of the groups (maximum 2 stars); and ascertainment of the outcome of interest (maximum 3 stars).

### Statistical analysis

Data from the included studies was pooled according to whether a complication after mesh implant occurred or not. The data was recorded in Microsoft Excel and analysed using Graphpad Prism 8.0 (Graphpad Software, Inc., La Jolla, CA).

## Results

**Fig. 1:**
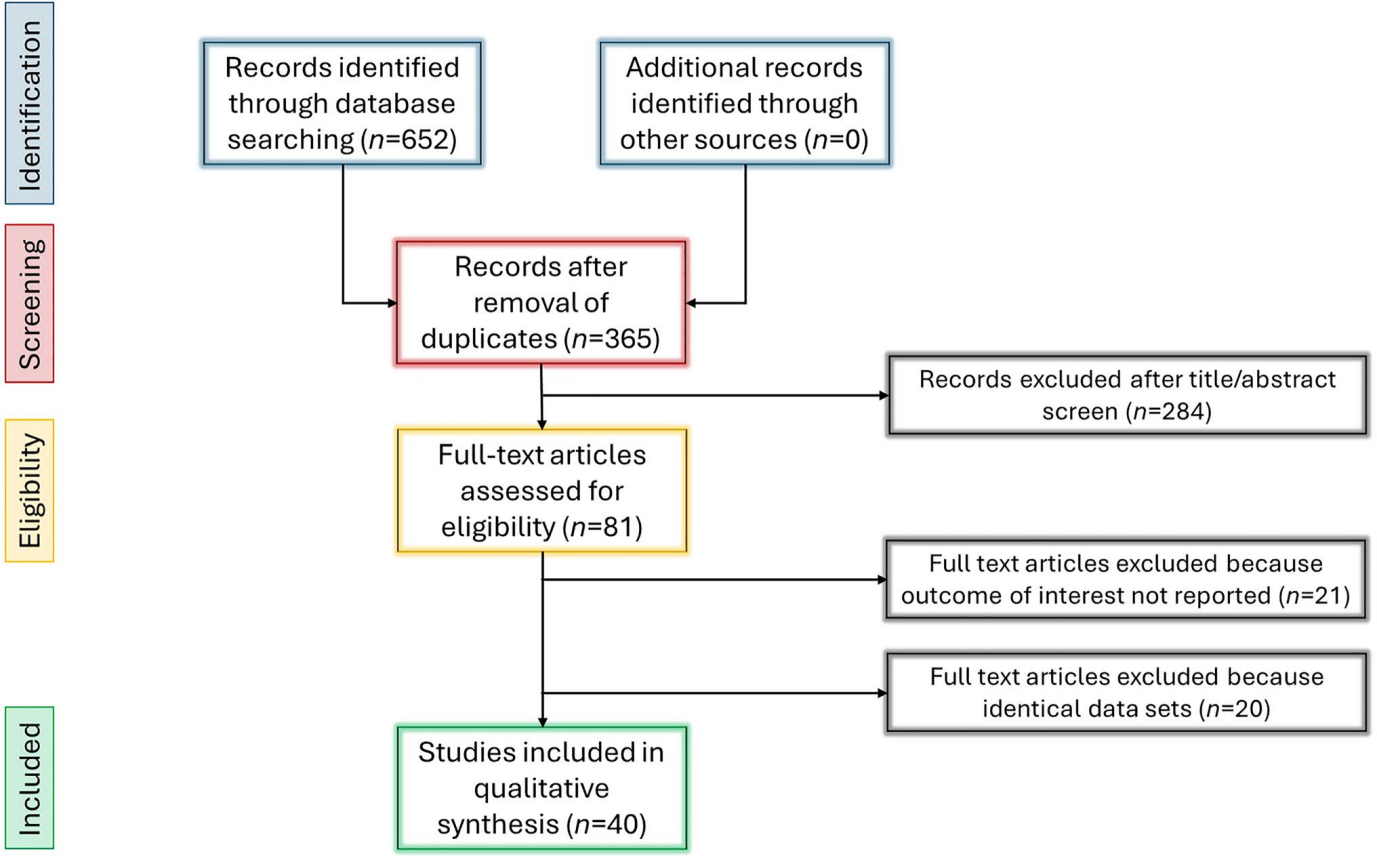
Flowchart

Figure 1 shows the flow chart with inclusion and exclusion criteria. Data extraction from electronic databases resulted in a total of 652 abstracts. After the exclusion of duplicates and non-relevant citations, 81 studies of potential relevance remained for full-text screening. Four studies were excluded, because outcome parameters of interest were either not reported or unavailable. Another 20 papers were excluded due to multiple use of the same data set(s). Studies were included based on the amount of data, the time period and the quality of the reported points of interest. Following screening, 40 studies were included for qualitative synthesis. A total of three RCTs with a cumulative number of114 VMR patients were found (Table 1) [19–21]. In 13 studies, a prospective design was present with 894 consecutive patients, (Table 2) [22–34]. A total of 24 retrospective studies [35–58] with 5261 patients were considered (Table 3). Regarding the operation approach, 5986 (95.5%) received a laparoscopic repair and 283 (4.5%) were operated with the robot. A redo operation was performed in 156 (2.5%) patients, whereas a combined operation (i.e. sacrocolpopexy), when documented by the authors, took place in 307 (4.9%) patients (Table 4).

**Table 1:** Randomised controlled trials (RCTs) included in the systematic review

	Country	*n*	Study group		Mesh complications		Total complications	
			LVMR *n* = 97	Control *n* = 79	LVMR (%)	Control (%)	LVMR (%)	Control (%)
Mehmood et al. [19]	UK	51	LVMR (*n* = 34)	Robotic (*n* = 17)	0	0	6 (18)	0
Lundby et al. [20]	Denmark	38	LVMR (*n* = 38)	LPSR (*n* = 37)	0	–*	2 (5)	1 (3)
Emile et al. [21]	Egypt	25	LVMR (*n* = 25)	Delorme (*n* = 25)	0	–*	5 (20)	3 (12)

*LVMR* laparoscopic ventral mesh rectopexy, *LPSR* laparoscopic posterior suture rectopexy

*No mesh in use

**Table 2:** Non-randomised, prospective studies included in the systematic review

	Country	Type of study	*n* = 894	Complications		Newcastle–Ottawa Scale		
				Mesh (%)	Total (%)	S	C	E
Wong et al. [22]	France	PCH	84	1 (1.1)	2 (2.4)	xx*x	*	*x*
Faucheron et al. [23]	France	PCH	175	1 (0.6)	7 (2.3)	*x**	**	***
van der Hagen et al. [24]	Netherlands	PCH	27	0	2 (7.4)	xxx*	x	*xx
Maggiori et al. [25]	France	PCH	33	0	0	xx*x	*	**x
Tsunoda et al. [26]	Japan	PCH	68	0	n.a	xx**	x	xxx
Ye et al. [27]	China	PCH	19	0	n.a	xxx*	x	*xx
Gurland et al. [28]	USA	CS	101	0	22 (31.0)	*x*x	*	**x
Abdulwahab et al. [29]	Egypt	PCH	33	0	3 (9.1)	xx*x	*	*xx
Gültekin et al. [30]	Turkey	CS	30	0	0	xxxx	x	**x
Postillon et al. [31]	France	PCH	96	0	12 (12.5)	*x**	**	***
Degasperi et al. [32]	Italy	PCH	50	0	1 (2.0)	*x*x	*	**x
Alemrajabi et al. [33]	Iran	PCH	156	0	6 (3.9)	*x**	*	***
Fabiani et al. [34]	Italy	PCH	22	0	0	xxx*	x	**x

*PCH* prospective cohort with historical control, *CS* case series, *PPS* point prevalence study

Newcastle–Ottawa Scale: S (****) 1. Representativeness of exposed cohort 2. Selection of the nonexposed cohort 3. Ascertainment of exposure 4. demonstration that outcome of interest was not present at the start of the study C (**) 5. Comparability of cohorts on the basis of the design or analysis E (***) 6. assessment of outcome 7. was follow-up long enough for outcome to occur? 8. adequacy of follow-up of cohorts

**Table 3:** Non-randomised, retrospective studies included in the systematic review

	Country	Type of study	*n* = 5261	Complications (%)		Newcastle–Ottawa Scale		
				Mesh	Total	S	C	E
van den Esschert et al. [35]	Netherlands	RCH	17	1 (5.8)	5 (35.3)	xxxx	x	*xx
Lauretta et al. [36]	Italy	RCH	30	0	1 (3.3)	xxxx	x	*xx
Formijne Jonkers et al. [37]	Netherlands	RCG	245	0	9 (3.7)	*x**	x	*x*
Ogilvie et al. [38]	USA	CMS	58	1 (1.7)	10 (17.2)	*x**	*	*x*
Consten et al. [39]	Belgium	multi RCH	919	18 (2.0)	114 (12.4)	*x**	x	**x
Evans et al. [40]	UK	RCH	2203	45 (2)	239 (10.8)	*x**	**	***
Horisberger et al. [41]	Switzerland/Germany	RCH	27	2 (7.4)	2 (7.4)	xxxx	x	*xx
Albayati et al. [42]	Australia	RCH	51	0	7 (13.7)	*x*x	x	*xx
Silveira et al. [43]	France/Brazil	RCH	176	1 (0.6)	13 (7.4)	*x**	*	*xx
Inaba et al. [44]	USA	RCH	24	0	0	xxxx	x	*xx
Fu et al. [45]	Singapore/Australia	RCH	231	1 (0.4)	17 (7.4)	*x**	x	*x*
Madbouly et al. [46]	Egypt	Case Series	41	1 (2.4)	6 (14.6)	*x**	*	*x*
Yang et al. [47]	Korea	RCH	69	0	3 (4.3)	*xxx	x	*xx
Gleditsch et al. [48]	Norway	RCH	22	0	2 (9.1)	*x**	x	**x
Mäkelä et al. [49]	Finland	RCH	501	7 (1.4)	33 (6.6)	****	x	*x*
Ahmad et al. [50]	UK	RCH	58	0	0	*xxx	x	*xx
Kremel et al. [51]	UK/Austria/Switzerland	RCH	74	2 (2.7)	14 (18.9)	*xx*	x	*xx
Brunner et al. [52]	Germany	RCH	123	0	17 (13.8)	*xx*	**	*x*
Altomare et al. [53]	Italy	RCH	21	0	1 (4.8)	xxx*	*	*x*
Chandra et al. [54]	India	RCH	25	0	6 (24)	xxxx	x	*xx
Martin del Olmo et al. [55]	Spain	RCH	32	1 (3.1)	1 (3.1)	xxxx	x	*xx
Campagna et al. [56]	Italy	RCH	98	0	2 (1.9)	*xx*	x	*xx
Tsiaousidou et al. [57]	UK	RCH	86	3 (3.5)	5 (5.8)	*xx*	*	*x*
Olatunbode et al. [58]	UK	RCH	130	3 (2.3)	4 (3.1)	*x**	*	*x*

*RCH* retrospective cohort with historical control, *RCG* research clinic group, *CMS* case-matched series, *CS* case series

Newcastle–Ottawa Scale: S (****) 1. Representativeness of exposed cohort 2. Selection of the nonexposed cohort 3. Ascertainment of exposure 4. demonstration that outcome of interest was not present at the start of the study C (**) 5. Comparability of cohorts on the basis of the design or analysis E (***) 6. assessment of outcome 7. was follow-up long enough for outcome to occur? 8. adequacy of follow-up of cohorts

**Table 4:** Operation details

	VMR *n* = 6269
Operation approach	
Laparoscopic ventral mesh rectopexy Robotic ventral mesh rectopexy	5986 (95.5%) 283 (4.5%)
Redo operation	156 (2.5%)
Combination procedure*	
Sacrocolpopexy Rectocolposacropexy Midurethral sling Posterior colporrhaphy Bladder mesh because of cystocele Hysterectomy Salpingectomy/oophorectomy Anterior vaginal mesh Rectocele repair Transvaginal tape	156 (2.5%) 32 (0.5%) 30 (0.5%) 27 (0.4%) 26 (0.4%) 15 (0.2%) 7 (0.1%) 7 (0.1%) 5 (0.1%) 2 (0.03%)

*VMR* ventral mesh rectopexy

*Reproducible combination interventions were described in six papers [22, 24, 28, 49, 55, 56]

### Mesh complications

**Fig. 2:**
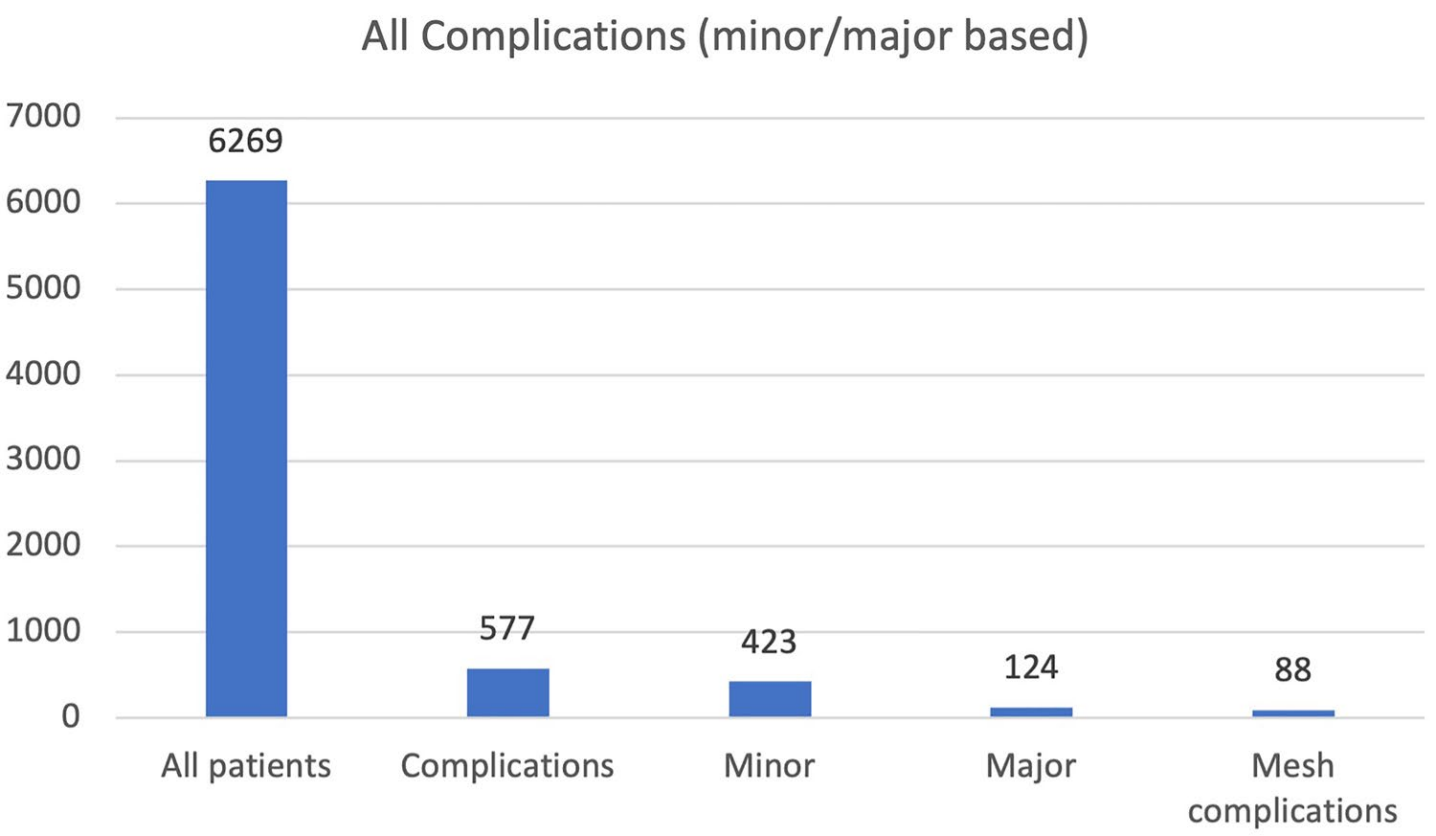
Complications

Across all studies, 577 complications were found in 6269 patients (9.2%). Among the complications, 423 (6.8%) were classified as minor and 124 (2.0%) as major In 21/40 (53%) studies, a distinction between major and minor complications was made by the author (Fig. 2).

Mesh-related complications were described in 88 patients (1.4%). The most common complication was mesh-erosion in 57 cases (64.8%), followed by rectovaginal fistula in 10 patients (11.4%) and 12 mesh releases (13.6%). Less common complications include discitis, pain and adhesions as well as of one complication not classified. The different complications are listed in Table 5. Only three studies stated postoperative mortality, with 5 patients in total (0.08%). Death from non-surgical causes were included. In general, the documentation of complications varied widely. While specific complications such as erosions, fistula and mesh release were commonly reported even if there was no such complication present, the standardised report of, for example pain, is questionable. Due to lack of standardised reporting of complications such as pain, limited information could be gained from this compilation.

**Table 5:** Mesh-related complications

	*n*	Mesh complications (*n* = 88)
Complication type		
Erosion^ç^ (specific)		
∙ Vaginal ∙ Rectal ∙ Perineal Erosion (unspecific)	25 18 1 13	28.4% 21.6% 1.1% 14.8%
Rectovaginal fistula^%^	10	11.4%
Mesh release^+^	12	13.6%
Discitis^&^	3	3.4%
Pain°	3	3.4%
Adhesion SB/Omentum^?^	2	2.3%
Unknown*	1	1.1%

*SB* small bowel

^ç^[23, 39, 40, 46, 49, 51, 55], ^%^[39, 40, 49], ^+^[39, 41, 43], ^&^[38, 45], °[51, 57], ^?^[35, 39], *[41]

### Mesh characteristics and fixation

A synthetic mesh was inserted in 2963 consecutive patients, while in 294 patients only biological meshes were used. The remaining 6223 patients were either from a study where both, synthetic and biological meshes were used or no specification about the mesh was made. Generally, mesh-related complications are low, ranging from 0.5% with biological mesh was used, up to 1.9% from studies where the mesh type was not specified. Despite being one of the top three most commonly reported complications, mesh release was not reported following use of biological mesh. Over a third of mesh erosions occurred following a synthetic mesh implant. The group where both types of meshes were used contained the most erosions and fistulations (Table 6).

**Table 6:** Reported mesh complications in relation to mesh type

	Cases	Complications	Erosion	Fistulation	Mesh release
Total	6269	88 (1.4%)	57 (0.9%)	10 (0.2%)	12 (0.2%)
Synthetic	2963	30 (1%)	15 (0.5%)	3 (0.1%)	10 (0.3%)
Biological	311	3 (1%)	0 (0%)	0 (0%)	0 (0%)
Both/unknown	2995	55 (1.8%)	42 (1.4%)	7 (0.2%)	2 (0.1%)

Regarding mesh fixation, the use of non-absorbable material was the favoured technique, followed by a combination of absorbable and non-absorbable material. Absorbable fixation alone was never used. Complication rates related to suture material were 1.5% or below (Table 7).

**Table 7:** Mesh complications in relation to mesh type and fixation material

	Synthetic (*n* = 2931)	Complication (*n* = 30)	Biological (*n* = 311)	Complication (*n* = 3)	Both (*n* = 542)	Complication (*n* = 5)
Absorbable	*–*	*–*	**–**	**–**	–	–
Non-absorb-able	*2109*	*21 (1%)*	**311**	**3 (1%)**	289	4 (1.4%)
Combination	*822*	*9 (1.1%)*	**–**	**–**	253	1 (0.4%)

Only the patients with defined mesh and fixation material were included. Synthetic mesh (italic), biological mesh (bold), synthetic and biological mesh (underline)

Complication rates due to fixation technique, are described in Table 8. Different fixation techniques included sutures alone or sutures in combination with a tacker. The highest complication rate of 2% among 2519 patients was observed in the non-specified mesh group when only sutures were used. In the synthetic mesh group, a single-technique appeared slightly superior to a combination of tacker and suture.

**Table 8:** Mesh complications in relation to mesh type and suture technique

	Synthetic (*n* = 2763)	Complication (*n* = 30)	Biological (*n* = 311)	Complication (*n* = 3)	Both (*n* = 2837)	Complication (*n* = 52)
Suture	*1103*	*10 (0.9%)*	**–**	**–**	2548	50 (2%)
Tacker	*232*	*1 (0.4%)*	**–**	**–**	–	–
Combination	*1428*	*19 (1.3%)*	**311**	**3 (1%)**	289	2 (0.7%)

Only the patients with defined mesh and suture technique were included. Synthetic mesh (italic), biological mesh (bold), synthetic and biological mesh (underline)

## Discussion

Our detailed analysis of the current literature has demonstrated that minimally invasive mesh rectopexy is a safe procedure with a low rate of mesh-associated complications but with a relevant number of overall morbidity of approximately 9%. Although fewer complications were observed with use of biological meshes a definitive statement cannot be made as this group was underrepresented in the studies considered.

There are a number of studies and systematic reviews describing the outcome and complications of the surgical treatment in rectopexy. Smart et al. discussed the difference between synthetic and biological mesh in their 2013 review, and found that the complication rate in both groups was low [12]. In 2019, a systematic review, meta-analysis and meta-regression by Emile et al. depicted laparoscopic VMR (LVMR) as a safe and effective option in full-thickness RP treatment. Additionally, they assumed that male patients and the length of the mesh potentially had an impact on the recurrence rate [59]. Recently, van der Schans et al. conducted a systematic review and meta-analysis describing the mesh-related complications with synthetic versus biological mesh [14]. Due to the heterogeneity and the quality of the study, they were not able to state a definitive conclusion. Regarding the frequency of mesh complications, Evans et al. analysed 2203 patients in their multicentre collaboration and found erosions in 45 patients, which is 2% (2.4% synthetic, 0.7% biological), with a re-operation rate over 90% [40]. From the initial 45 erosions, three patients had a mesh removal with colostomy and three an ultra-low anterior resection with a temporary ileostomy [40]. Balla et al. analysed 8 studies in their systematic review in 2017 and mentioned an erosion rate for synthetic and biological meshes of 1.87 and 0.22%, respectively [13]. All erosions were treated surgically [13]. Our results of 0.9% erosion overall are thus in line with the current literature. A release of the mesh was one of the frequently reported mesh complications. The clinical relevance can, depending on the time of occurrence, be of crucial importance. An inadequate fixation, whether through absorbable or non-absorbable material, can be of utmost importance with regard to an early recurrence. This is because, regardless of the mesh material used, these complications occur early and the resorption of a biological mesh may not yet have fully taken place. Moreover, a loss of mesh-continuity, if not actively sought, is potentially under-represented in asymptomatic patients.

Generally, the documentation of adverse events across the studies included varied widely. This could be related to the consideration that VMR is a low risk operation, therefore there is a variety of granularity concerning the reporting of complications. The focus is often on recurrence and generally the clinical outcome with improvement of the underlying cause for surgery. A simple factor like pain after surgery is documented as complication in only two studies [38, 51].

In this review of 40 studies reporting the mesh complications after (L/R)VMR only 1.4% adverse events were stated. This rate is in line with the recently published data of van der Schans et al. which showed complications for synthetic and biological meshes up to 2.4 and 0.7% respectively [14]. Although it appears, that a biological mesh should be favoured in terms of mesh-related complications, van der Schans et al. could not find any literature supporting a clear statement in favour of any one mesh type [14]. Our data confirms the low complication rate of biological, compared to synthetic meshes. The recurrence rate is one of the significant points regarding the LVMR, but no statement can be made in this regard with our data. In their systematic review 2017, Balla et al. analysed the erosion rates after LVMR, with significantly more erosions after insertion of a synthetic mesh [13].

The complication of mesh release is also rare, but of great therapeutic relevance if detected, as mentioned above. Consten et al. described in their cohort of 919 patients 9 such detachments, with consecutive re-operation Consten et al. [39]. In biological mesh repair, a mesh release logically has a much lower or no significance due to the resorbability of the mesh. Due to this, potentially high number of unreported cases of mesh release should also be considered.

The placement of a synthetic mesh can be associated with fistulation or the occurrence of erosions while these risks are reduced through the use of a biological mesh [12, 13]. Our data described a much higher erosion and fistulation rate in the synthetic mesh group, while no erosion or fistulation was observed in biological mesh patients. The position statement by the Pelvic Floor Society also states that synthetic meshes are related to higher morbidity [60]. An erosion often results in a surgical re-intervention, but interestingly, the time of diagnosis can vary widely [13].

The interpretation of the results is affected by two other potentially relevant factors. In the data considered, redo-operations were carried out, albeit in small numbers. Furthermore, a few authors documented combination surgeries. Both issues hamper the inclusive assessment of complication rates. However, the documented redo operations are at 2.5% and the combination operations cumulatively at less than 5%. This data would have to be analysed separately for a reasonable assessment.

In addition to the mesh type, the material and technique used are also discussed in literature. Only a few patients had their fixation through solely absorbable sutures, while the vast majority had non-absorbable fixation or a combination of absorbable and non-absorbable suture fixation. Most often, the technique contained a combination of tacker and suture fixation. The literature is currently vague regarding a material recommendation. Mercer-Jones et al. comments on the possible influence of the suture material on morbidity in their position statement [60]. Tejedor et al. analysed 495 patients in a matched-case study and found a 3.3% erosion-rate after the use of non-absorbable material. Meanwhile, there were no complications in the absorbable group [61]. In a large gynaecological systematic review about laparoscopic sacrocolpopexy, conducted by Gluck et al., the topic of absorbable and non-absorbable sutures was discussed briefly [62]. They stated no difference in mesh failure when late-absorbable material was used (level 2 of evidence) and also glue appeared to be safe (level 3 of evidence) [62].

Uniform, large-scale, randomised studies are still needed to make a definitive recommendation regarding mesh selection. To date, biological mesh has been used in patients at risk of fistula, possibly because the higher costs are thought to be justified given the assumed lower mesh-associated fistula rate. However, there seems to be a trend towards a slightly higher risk of recurrence [14].

We would like to acknowledge the limitations of our study: First, this is a retrospective analysis with the potential risk of lack of information and loss to follow-up. Secondly, documentation of the (mesh) complications may be under-reported. Thirdly, a clear definition of complication and mesh-related complication was sometimes difficult to set. And lastly, the data suffers from huge heterogeneity.

In conclusion, rectopexy, especially the laparoscopic procedure with mesh insertion, is a well established and safe operation with a low risk of mesh-related complications.

## References

[1] Kairaluoma MV, Kellokumpu IH (2005) Epidemiologic aspects of complete rectal prolapse. Scand J Surg 94:207–210 16259169 10.1177/145749690509400306

[2] Murad-Regadas SM, Pinto RA (2016) Treatment of rectal prolapse. Semin Colon Rectal Surg 27(1):33–39

[3] Adusumilli S, Gosselink MP, Fourie S, Curran K, Jones OM, Cunningham C, Lindsey I (2013) Does the presence of a high grade internal rectal prolapse affect the outcome of pelvic floor retraining in patients with faecal incontinence or obstructed defaecation? Colorectal Dis 15:e680–e685 23890098 10.1111/codi.12367

[4] D’Hoore A, Cadoni R, Penninckx F (2004) Long-term outcome of laparoscopic ventral rectopexy for total rectal prolapse. Br J Surg 91:1500–1505 15499644 10.1002/bjs.4779

[5] Faucheron J-L, Trilling B, Girard E, Sage P-Y, Barbois S, Reche F (2015) Anterior rectopexy for full-thickness rectal prolapse: technical and functional results. World J Gastroenterol 21:5049–5055 25945021 10.3748/wjg.v21.i16.5049 PMC4408480

[6] Baker R, Senagore AJ, Luchtefeld MA (1995) Laparoscopic-assisted vs. open resection. Rectopexy offers excellent results. Dis Colon Rectum 38:199–201 7851177 10.1007/BF02052451

[7] Kairaluoma MV, Viljakka MT, Kellokumpu IH (2003) Open vs. laparoscopic surgery for rectal prolapse: a case-controlled study assessing short-term outcome. Dis Colon Rectum 46:353–360 12626911 10.1007/s10350-004-6555-8

[8] Purkayastha S, Tekkis P, Athanasiou T, Aziz O, Paraskevas P, Ziprin P, Darzi A (2005) A comparison of open vs. laparoscopic abdominal rectopexy for full-thickness rectal prolapse: a meta-analysis. Dis Colon Rectum 48:1930–1940 15981060 10.1007/s10350-005-0077-x

[9] Byrne CM, Smith SR, Solomon MJ, Young JM, Eyers AA, Young CJ (2008) Long-term functional outcomes after laparoscopic and open rectopexy for the treatment of rectal prolapse. Dis Colon Rectum 51:1597–1604 18758861 10.1007/s10350-008-9365-6

[10] NICE (2018) Laparoscopic ventral mesh rectopexy for internal rectal prolapse. Interventional procedures guidance [IPG618]

[11] U.S. Food & Drug Administration (2021) Pelvic organ prolapse (POP): surgical mesh considerations and recommendations. https://www.fda.gov/medical-devices/urogynecologic-surgical-mesh-implants/pelvic-organ-prolapse-pop-surgical-mesh-considerations-and-recommendations. Accessed 28 Oct 2023

[12] Smart NJ, Pathak S, Boorman P, Daniels IR (2013) Synthetic or biological mesh use in laparoscopic ventral mesh rectopexy—a systematic review. Colorectal Dis 15:650–654 23517144 10.1111/codi.12219

[13] Balla A, Quaresima S, Smolarek S, Shalaby M, Missori G, Sileri P (2017) Synthetic versus biological mesh-related erosion after laparoscopic ventral mesh rectopexy: a systematic review. Ann Coloproctol 33:46–51 28503515 10.3393/ac.2017.33.2.46 PMC5426201

[14] van der Schans EM, Boom MA, El Moumni M, Verheijen PM, Broeders IAMJ, Consten ECJ (2022) Mesh-related complications and recurrence after ventral mesh rectopexy with synthetic versus biologic mesh: a systematic review and meta-analysis. Tech Coloproctol 26:85–98 34812970 10.1007/s10151-021-02534-4 PMC8763765

[15] Liberati A, Altman DG, Tetzlaff J, Mulrow C, Gøtzsche PC, Ioannidis JPA, Clarke M, Devereaux PJ, Kleijnen J, Moher D (2009) The PRISMA statement for reporting systematic reviews and meta-analyses of studies that evaluate health care interventions: explanation and elaboration. J Clin Epidemiol 62:e1-34 19631507 10.1016/j.jclinepi.2009.06.006

[16] Dindo D, Demartines N, Clavien P-A (2004) Classification of surgical complications: a new proposal with evaluation in a cohort of 6336 patients and results of a survey. Ann Surg 240:205 15273542 10.1097/01.sla.0000133083.54934.ae PMC1360123

[17] Cumpston M, Li T, Page MJ, Chandler J, Welch VA, Higgins JP, Thomas J (2019) Updated guidance for trusted systematic reviews: a new edition of the Cochrane handbook for systematic reviews of interventions. Cochrane Database Syst Rev 10:ED000142 10.1002/14651858.ED000142 PMC10284251 31643080

[18] Wells GA, Shea B, O’Connell D, Peterson J,Welch V, Losos M et al. (n.d.) The Newcastle–Ottawa Scale for assessing the quality of nonrandomised studies in meta-analyses

[19] Mehmood RK, Parker J, Bhuvimanian L, Qasem E, Mohammed AA, Zeeshan M, Grugel K, Carter P, Ahmed S (2014) Short-term outcome of laparoscopic versus robotic ventral mesh rectopexy for full-thickness rectal prolapse. Is robotic superior? Int J Colorectal Dis 29:1113–1118 24965859 10.1007/s00384-014-1937-4

[20] Lundby L, Iversen LH, Buntzen S, Wara P, Høyer K, Laurberg S (2016) Bowel function after laparoscopic posterior sutured rectopexy versus ventral mesh rectopexy for rectal prolapse: a double-blind, randomised single-centre study. Lancet Gastroenterol Hepatol 1:291–297 28404199 10.1016/S2468-1253(16)30085-1

[21] Emile SH, Elbanna H, Youssef M, Thabet W, Omar W, Elshobaky A, Abd El-Hamed TM, Farid M (2017) Laparoscopic ventral mesh rectopexy vs Delorme’s operation in management of complete rectal prolapse: a prospective randomized study. Colorectal Dis 19:50–57 27225971 10.1111/codi.13399

[22] Wong M, Meurette G, Abet E, Podevin J, Lehur PA (2011) Safety and efficacy of laparoscopic ventral mesh rectopexy for complex rectocele. Colorectal Dis 13:1019–1023 20553314 10.1111/j.1463-1318.2010.02349.x

[23] Faucheron J-L, Voirin D, Riboud R, Waroquet P-A, Noel J (2012) Laparoscopic anterior rectopexy to the promontory for full-thickness rectal prolapse in 175 consecutive patients: short- and long-term follow-up. Dis Colon Rectum 55:660–665 22595845 10.1097/DCR.0b013e318251612e

[24] van der Hagen SJ, van Gemert WG, Soeters PB, de Wet H, Baeten CG (2012) Transvaginal posterior colporrhaphy combined with laparoscopic ventral mesh rectopexy for isolated Grade III rectocele: a prospective study of 27 patients. Colorectal Dis 14:1398–1402 22405411 10.1111/j.1463-1318.2012.03023.x

[25] Maggiori L, Bretagnol F, Ferron M, Panis Y (2013) Laparoscopic ventral rectopexy: a prospective long-term evaluation of functional results and quality of life. Tech Coloproctol 17:431–436 23345041 10.1007/s10151-013-0973-3

[26] Tsunoda A, Takahashi T, Ohta T, Kusanagi H (2016) A novel technique of introducing the mesh at the distal dissection while performing laparoscopic ventral rectopexy. Colorectal Dis 18:O334–O336 27427829 10.1111/codi.13463

[27] Ye GY, Wang Z, Matzel KE, Cui Z (2017) Short-term outcomes of laparoscopic ventral rectopexy for obstructed defecation in patients with overt pelvic structural abnormalities-a Chinese pilot study. Int J Colorectal Dis 32:1337–1340 28409269 10.1007/s00384-017-2815-7

[28] Gurland BE, Carvalho MEC, Ridgeway B, Paraiso MFR, Hull T, Zutshi M (2017) Should we offer ventral rectopexy to patients with recurrent external rectal prolapse? Int J Colorectal Dis 32:1561–1567 28785819 10.1007/s00384-017-2858-9

[29] Abdulwahab MB, Elgohary H (2017) Short-term surgical and functional outcome of laparoscopic ventral mesh rectopexy for management of complete rectal prolapse. Egyptian J Surg 36:208

[30] Gültekin FA (2019) Short term outcome of laparoscopic ventral mesh rectopexy for rectal and complex pelvic organ prolapse: case series. Turk J Surg 35:91–97 32550312 10.5578/turkjsurg.4157 PMC6796075

[31] Postillon A, Perrenot C, Germain A, Scherrer M-L, Buisset C, Brunaud L, Ayav A, Bresler L (2020) Long-term outcomes of robotic ventral mesh rectopexy for external rectal prolapse. Surg Endosc 34:930–939 31183789 10.1007/s00464-019-06851-6

[32] Degasperi S, Scarpa M, Zini O, Ruffolo C, Gruppo M, Bardini R, Angriman I (2020) Laparoscopic ventral rectopexy for obstructed defecation: functional results and quality of life. Surg Laparosc Endosc Percutan Tech 31:14–19 32740474 10.1097/SLE.0000000000000835

[33] Alemrajabi M, Darabi B, Banivaheb B, Hemmati N, Jahanian S, Moradi M (2020) Polyvinylidene fluoride mesh use in laparoscopic ventral mesh rectopexy in patients with obstructive defecation syndrome for the first time. J Invest Surg 34:1–6 10.1080/08941939.2020.1767734 32423243

[34] Fabiani B, Sturiale A, Fralleone L, Menconi C, d’Adamo V, Naldini G (2023) Modified robotic ventral rectopexy with folded single titanized mesh suspension for the treatment of complex pelvic organ prolapse. Colorectal Dis 25:453–457 36200305 10.1111/codi.16351

[35] van den Esschert JW, van Geloven AAW, Vermulst N, Groenedijk AG, de Wit LT, Gerhards MF (2008) Laparoscopic ventral rectopexy for obstructed defecation syndrome. Surg Endosc 22:2728–2732 18320283 10.1007/s00464-008-9771-9

[36] Lauretta A, Bellomo RE, Galanti F, Tonizzo CA, Infantino A (2012) Laparoscopic low ventral rectocolpopexy (LLVR) for rectal and rectogenital prolapse: surgical technique and functional results. Tech Coloproctol 16:477–483 23104551 10.1007/s10151-012-0918-2

[37] Formijne Jonkers HA, Poierrié N, Draaisma WA, Broeders IAMJ, Consten ECJ (2013) Laparoscopic ventral rectopexy for rectal prolapse and symptomatic rectocele: an analysis of 245 consecutive patients. Colorectal Dis 15:695–699 23406289 10.1111/codi.12113

[38] Ogilvie JW Jr, Stevenson ARL, Powar M (2014) Case-matched series of a non-cross-linked biologic versus non-absorbable mesh in laparoscopic ventral rectopexy. Int J Colorectal Dis 29:1477–1483 25310924 10.1007/s00384-014-2016-6

[39] Consten ECJ, van Iersel JJ, Verheijen PM, Broeders IAMJ, Wolthuis AM, D’Hoore A (2015) Long-term outcome after laparoscopic ventral mesh rectopexy: an observational study of 919 consecutive patients. Ann Surg 262:742–747 26583661 10.1097/SLA.0000000000001401

[40] Evans C, Stevenson ARL, Sileri P, Mercer-Jones MA, Dixon AR, Cunningham C, Jones OM, Lindsey I (2015) A multicenter collaboration to assess the safety of laparoscopic ventral rectopexy. Dis Colon Rectum 58:799–807 26163960 10.1097/DCR.0000000000000402

[41] Horisberger K, Rickert A, Templin S, Post S, Kienle P (2016) Laparoscopic ventral mesh rectopexy in complex pelvic floor disorder. Int J Colorectal Dis 31:991–996 27041555 10.1007/s00384-016-2545-2

[42] Albayati S, Morgan MJ, Turner CE (2017) Laparoscopic ventral rectopexy for rectal prolapse and rectal intussusception using a biological mesh. Colorectal Dis 19:857–862 28371010 10.1111/codi.13671

[43] Silveira RK, Domingie S, Kirzin S, de Melo Filho DA, Portier G (2017) Comparative study of safety and efficacy of synthetic surgical glue for mesh fixation in ventral rectopexy. Surg Endosc 31:4016–4024 28364153 10.1007/s00464-017-5439-7

[44] Inaba CS, Sujatha-Bhaskar S, Koh CY, Jafari MD, Mills SD, Carmichael JC, Stamos MJ, Pigazzi A (2017) Robotic ventral mesh rectopexy for rectal prolapse: a single-institution experience. Tech Coloproctol 21:667–671 28871416 10.1007/s10151-017-1675-z

[45] Fu CWP, Stevenson ARL (2017) Risk factors for recurrence after laparoscopic ventral rectopexy. Dis Colon Rectum 60:178–186 28059914 10.1097/DCR.0000000000000710

[46] Madbouly KM, Youssef M (2018) Laparoscopic ventral rectopexy versus laparoscopic wells rectopexy for complete rectal prolapse: long-term results. J Laparoendosc Adv Surg Tech A 28:1–6 28586260 10.1089/lap.2017.0012

[47] Yang S-J, Yoon S-G, Lim K-Y, Lee J-K (2017) Laparoscopic vaginal suspension and rectopexy for rectal prolapse. Ann Coloproctol 33:64–69 28503518 10.3393/ac.2017.33.2.64 PMC5426198

[48] Gleditsch D, Wexels WA, Nesbakken A (2018) Surgical options and trends in treating rectal prolapse: long-term results in a 19-year follow-up study. Langenbecks Arch Surg 403:991–998 30415286 10.1007/s00423-018-1728-4

[49] Mäkelä-Kaikkonen J, Rautio T, Kairaluoma M, Carpelan-Holmström M, Kössi J, Rautio A, Ohtonen P, Mäkelä J (2018) Does ventral rectopexy improve pelvic floor function in the long term? Dis Colon Rectum 61:230–238 29337779 10.1097/DCR.0000000000000974

[50] Ahmad NZ, Stefan S, Adukia V, Naqvi SAH, Khan J (2018) Laparoscopic ventral mesh rectopexy: functional outcomes after surgery. Surg J 4:e205–e211 10.1055/s-0038-1675358 PMC6205861 30377654

[51] Kremel D, Riss S, Müller C, von Strauss M, Winstanley C, Winstanley J, Potter M, Paterson H, Collie M (2018) Adverse obstetric history is not a risk factor for poor outcome after ventral rectopexy for obstructive defaecation syndrome. Colorectal Dis 20:1125–1131 30171744 10.1111/codi.14392

[52] Brunner M, Roth H, Günther K, Grützmann R, Matzel KE (2018) Ventral rectopexy with biological mesh: short-term functional results. Int J Colorectal Dis 33:449–457 29442156 10.1007/s00384-018-2972-3

[53] Altomare DF, Picciariello A, Memeo R, Fanelli M, Digennaro R, Chetta N, De Fazio M (2018) Pelvic floor function following ventral rectopexy versus STARR in the treatment of obstructed defecation. Tech Coloproctol 22:289–294 29594747 10.1007/s10151-018-1776-3

[54] Chandra A, Singh P, Kumar S, Chopra N, Gupta V, Joshi P, Gupta V (2018) Laparoscopic ventral rectopexy: a viable option in procidentia with redundant sigmoid—an Indian perspective. J Minim Access Surg 14:304–310 29582793 10.4103/jmas.JMAS_106_17 PMC6130185

[55] Martín Del Olmo JC, Toledano M, Martín Esteban ML, Montenegro MA, Gómez JR, Concejo P, Rodríguez de Castro M, Del Rio F (2019) Outcomes of laparoscopic management of multicompartmental pelvic organ prolapse. Surg Endosc 33:1075–1079 29998390 10.1007/s00464-018-6357-z

[56] Campagna G, Panico G, Caramazza D, Anchora LP, Parello A, Gallucci V, Vacca L, Scambia G, Ercoli A, Ratto C (2020) Laparoscopic sacrocolpopexy plus ventral rectopexy as combined treatment for multicompartment pelvic organ prolapse. Tech Coloproctol 24:573–584 32285229 10.1007/s10151-020-02199-5

[57] Tsiaousidou A, MacDonald L, Shalli K (2022) Mesh safety in pelvic surgery: our experience and outcome of biological mesh used in laparoscopic ventral mesh rectopexy. World J Clin Cases 10:891–898 35127904 10.12998/wjcc.v10.i3.891 PMC8790465

[58] Olatunbode O, Rangarajan S, Russell V, Viswanath Y, Reddy A (2022) A quantitative study to explore functional outcomes following laparoscopic ventral mesh rectopexy for rectal prolapse. Ann R Coll Surg Engl 104:449–455 34939835 10.1308/rcsann.2021.0212 PMC9158073

[59] Emile SH, Elfeki H, Shalaby M, Sakr A, Sileri P, Wexner SD (2019) Outcome of laparoscopic ventral mesh rectopexy for full-thickness external rectal prolapse: a systematic review, meta-analysis, and meta-regression analysis of the predictors for recurrence. Surg Endosc. 10.1007/s00464-019-06803-0 31041515 10.1007/s00464-019-06803-0

[60] Mercer-Jones MA, Brown SR, Knowles CH, Williams AB (2020) Position statement by the pelvic floor society on behalf of the association of coloproctology of Great Britain and Ireland on the use of mesh in ventral mesh rectopexy. Colorectal Dis 22:1429–1435 28926174 10.1111/codi.13893 PMC7702115

[61] Tejedor P, Lindsey I, Jones OM, Jones HJS, Gorissen K, Penna M, Cunningham C (2019) Impact of suture type on erosion rate after laparoscopic ventral mesh rectopexy: a case-matched study. Dis Colon Rectum 62:1512–1517 31569096 10.1097/DCR.0000000000001510

[62] Gluck O, Blaganje M, Veit-Rubin N, Phillips C, Deprest J, O’reilly B, But I, Moore R, Jeffery S, Haddad JM, Deval B (2020) Laparoscopic sacrocolpopexy: a comprehensive literature review on current practice. Eur J Obstet Gynecol Reprod Biol 245:94–101 31891897 10.1016/j.ejogrb.2019.12.029

